# Prevalence of *Streptococcus pneumoniae* in conjunctival flora and association with nasopharyngeal carriage among children in a Vietnamese community

**DOI:** 10.1038/s41598-020-79175-4

**Published:** 2021-01-11

**Authors:** Yasser Helmy Mohamed, Michiko Toizumi, Masafumi Uematsu, Hien-Anh Thi Nguyen, Lien Thuy Le, Mizuki Takegata, Chihiro Iwasaki, Noriko Kitamura, Monica L. Nation, Eileen M. Dunne, Jason Hinds, Hung Thai Do, Mai Quang Vien, Catherine Satzke, Stefan Flasche, Kim Mulholland, Duc-Anh Dang, Takashi Kitaoka, Lay-Myint Yoshida

**Affiliations:** 1 Department of Ophthalmology and Visual Sciences, Graduate School of Biomedical Sciences, Nagasaki University, Nagasaki, Japan; 2 Department of Pediatric Infectious Diseases, Institute of Tropical Medicine, Nagasaki University, 1-12-4 Sakamoto, Nagasaki, 852-8523 Japan; 3 National Institute of Hygiene and Epidemiology, Hanoi, Vietnam; 4 Pasteur Institute in Nha Trang, Nha Trang, Vietnam; 5 Infection and Immunity, Murdoch Children’s Research Institute, Parkville, VIC Australia; 6 Institute for Infection and Immunity, St. George’s, University of London, London, UK; 7 London Bioscience Innovation Centre, BUGS Bioscience, London, UK; 8 Department of Paediatrics, The University of Melbourne, Parkville, VIC Australia; 9 Department of Microbiology and Immunology, The University of Melbourne at the Peter Doherty Institute for Infection and Immunity, Parkville, VIC Australia; 10 Department of Infectious Disease Epidemiology, London School of Hygiene and Tropical Medicine, London, UK

**Keywords:** Bacterial infection, Epidemiology, Conjunctival diseases

## Abstract

Conjunctival pneumococcal serotypes among members of a community have not been investigated well. We determined the prevalence and association of *Streptococcus pneumoniae* in the nasopharynx and conjunctiva among children in a community before pneumococcal conjugate vaccine introduction. In October 2016, conjunctival and nasopharyngeal swabs were collected from children (< 24 months old) and nasopharyngeal swabs from mothers in Nha Trang, Vietnam. Quantitative *lytA* PCR and DNA microarray were performed to detect and serotype *S. pneumoniae*. The association between *S. pneumoniae* in the nasopharynx and conjunctiva was evaluated using multivariable logistic regression model. Among 698 children, 62 (8.9%, 95% CI 6.9–11.2%) were positive for *S. pneumoniae* in the conjunctiva. Non-encapsulated *S. pneumoniae* were most commonly identified, followed by serotypes 6A, 6B, and 14. Nasopharyngeal and conjunctival detection were positively associated (aOR 47.30, 95% CI 24.07–92.97). Low birth-weight, day-care attendance, and recent eye symptoms were independently associated with *S. pneumoniae* detection in the conjunctiva (aOR 11.14, 95% CI 3.76–32.98, aOR 2.19, 95% CI 1.45–3.31, and aOR 3.59, 95% CI 2.21–5.84, respectively). Serotypes and genotypes in the conjunctiva and nasopharynx matched in 87% of the children. Three mothers’ nasopharyngeal pneumococcal samples had matched serotype and genotype with their child’s in the conjunctiva and nasopharynx. *S. pneumoniae* presence in nasopharynx and conjunctiva were strongly associated. The high concordance of serotypes suggests nasopharyngeal carriage may be a source of transmission to the conjunctiva.

## Introduction

The conjunctiva is commonly colonized by a diverse range of microorganisms that constitute the normal ocular flora. However, these microorganisms are also capable of causing infections of the conjunctiva and cornea^1,2^. Acute conjunctivitis is one of the most common ocular disorders among children younger than 6 years old^3^. Acute bacterial conjunctivitis accounts for approximately 50–75% of all conjunctivitis cases^4,5^. *Streptococcus pneumoniae* is one of the top three most commonly isolated microorganisms from normal conjunctival flora^6^. The reported prevalence of *S. pneumoniae* in healthy conjunctiva ranges from 0 to 4.2%^6–9^.

*S. pneumoniae* kills an estimated 317,300 children aged 1–59 months, mostly in lower income countries^10^. *S. pneumoniae* causes meningitis^11^, pneumonia^12^, otitis media^13^, sinusitis^14^, and acute conjunctivitis^15^. *S. pneumoniae* colonization in the nasopharynx is a prerequisite for pneumococcal disease and for transmission^16,17^. The prevalence of *S. pneumoniae* carriage in the nasopharynx increases in the first few years of life, peaking at approximately 50–80% in children 2–3 years of age, and decreasing thereafter until stabilizing at 5–10% in children over 10 years of age^16^. Overcrowding (as defined by family size) and day-care attendance are well-established factors associated with nasopharyngeal carriage of *S. pneumoniae*^18,19^.

*S. pneumoniae* is responsible for 7–44% of acute conjunctivitis and for 12–20% of conjunctivitis-otitis syndrome^20,21^. Previous studies found that non-typeable (NT) pneumococci were most likely to cause acute conjunctivitis^22,23^.

We did not find any previously published studies describing the pneumococcal serotypes detected in the conjunctiva among members of a community; furthermore, the association between *S. pneumoniae* prevalence in the conjunctiva and the nasopharynx has not been examined yet. We hypothesized that *S. pneumoniae* in these sites would be positively associated, because they are physically connected via the nasolacrimal duct, enabling bacterial transfer between the two niches. The objectives of this study were to determine the prevalence and serotypes of *S. pneumoniae* in conjunctival flora in children aged 4–23 months before the introduction of pneumococcal conjugate vaccine (PCV), and to investigate whether *S. pneumoniae* in the conjunctival flora is associated with nasopharyngeal carriage and other host factors. In addition, this study sought to determine whether the serotype in the conjunctiva was similar to that detected in the nasopharynx for each participant.

## Results

### Participants

Six hundred and ninety-eight children aged 4–23 months were enrolled, including six children aged 12–13 months. Although the six children aged 12–13 months were not eligible for the wider PCV study, we included them in the current study and categorized them in the group aged 12–23 months.

Among the 698 children, 54.2% (n = 378) were boys and the median age at examination was 11.7 months (interquartile range, 8.2–17.6). Ninety-nine percent of the children (n = 691) had never had PCV vaccination. Conjunctival and nasopharyngeal swabs were collected from all 698 enrolled children. Nasopharyngeal swabs were collected from all the 698 mothers of the children.

### Prevalence and factors associated with *S. pneumoniae* in the conjunctiva

Sixty-two children (8.9%, 95% CI 6.9–11.2%) had *S. pneumoniae* in the conjunctiva. Social demographics, clinical characteristics, and *S. pneumoniae* carriage in the child’s and mother’s nasopharynx among children with and without conjunctival *S. pneumoniae* are shown in Table 1. Respiratory hospitalization history, cough, runny nose, and eye symptoms in the last 2 weeks, day-care attendance, generally in the company of other children under five, and *S. pneumoniae* carriage in the child’s and the mother’s nasopharynx were positively associated with *S. pneumoniae* positive conjunctiva by univariable analysis.

**Table 1:** Effect of each characteristic on having *S. pneumoniae* in conjunctiva, estimated using logistic regression models.

Characteristics	*S. pneumoniae* positive conjunctiva N (%)/mean (SD) (n = 62)	*S. pneumoniae* negative conjunctiva N (%)/mean (SD) (n = 636)	Crude odds ratio of having *S. pneumoniae* in conjunctiva (95% CI)	Adjusted odds ratio^a^ of having *S. pneumoniae* in conjunctiva (95% CI)
**Demographics**				
Sex				
Girl	34 (10.6)	286 (89.4)	Reference	Reference
Boy	28 (7.4)	350 (92.6)	0.67 (0.40–1.14)	0.61 (0.26–1.43)
Age				
< 12 months	24 (6.8)	328 (93.2)	Reference	Reference
12–23 months	38 (11.0)	308 (89.0)	1.69 (0.99–2.88)	0.84 (0.65–1.08)
**Medical history**				
Birth weight				
≥ 2500 g	58 (8.5)	622 (91.5)	Reference	Reference
< 2500 g (low birth weight)	4 (22.2)	14 (77.8)	3.06 (0.98–9.61)	11.14 (3.76–32.98)
Gestational age at birth				
37 weeks or more	57 (8.7)	602 (91.4)	Reference	
< 37 weeks (preterm)	5 (12.8)	34 (87.2)	1.55 (0.58–4.13)	
Hospitalization for respiratory disease				
Yes	11 (20.8)	42 (79.3)	3.05 (1.48–6.28)	
No	51 (7.9)	594 (92.1)	Reference	
Pneumococcal conjugate vaccine history				
Yes (at least one dose)	0 (0.0)	7 (100.0)	Not examined	
No	62 (9.0)	629 (91.0)	Not examined	
**Child's symptom in last 2 weeks**				
Cough				
Yes	37 (11.9)	274 (88.1)	1.96 (1.15–3.33)	0.73 (0.32–1.63)
No	25 (6.5)	362 (93.5)	Reference	Reference
Runny nose				
Yes	41 (12.2)	295 (87.8)	2.26 (1.30–3.91)	1.08 (0.39–3.00)
No	21 (5.8)	341 (94.2)	Reference	Reference
Eye symptom				
Yes	10 (30.3)	23 (69.7)	5.13 (2.32–11.34)	3.59 (2.21–5.84)
No	52 (7.8)	613 (92.2)	Reference	Reference
**Breastfeeding and environment**				
Breastfeeding until 6 month (including mixed breastfeeding)				
Yes	58 (9.7)	540 (90.3)	2.58 (0.91–7.27)	
No	4 (4.0)	96 (96.0)	Reference	
Current breastfeeding				
Yes	37 (9.1)	371 (90.9)	1.06 (0.62–1.80)	
No	25 (8.6)	265 (91.4)	Reference	
Day-care attendance				
Yes	31 (19.4)	129 (80.6)	3.93 (2.30–6.70)	2.19 (1.45–3.31)
No	31 (5.8)	507 (94.2)	Reference	Reference
Generally in company with children < 5 years old				
Yes	53 (10.7)	444 (89.3)	2.55 (1.23–5.27)	
No	9 (4.5)	192 (95.5)	Reference	
**Socioeconomic status**				
Number of family member	5.5 (2.2)	5.5 (2.0)	1.02 (0.90–1.15)	1.08 (0.94–1.23)
Education level of mother				
Elementary school or lower	7 (10.1)	62 (89.9)	Reference	
Secondary school	9 (5.7)	150 (94.3)	0.53 (0.19–1.49)	
High school	18 (8.6)	192 (91.4)	0.83 (0.33–2.08)	
College, university or higher	28 (10.8)	232 (89.2)	1.07 (0.45–2.56)	
Household income last month (million Vietnamese dong)	12.6 (6.3)	11.7 (7.3)	1.02 (0.98–1.05)	
Smoker in household				
Yes	35 (8.5)	377 (91.5)	0.89 (0.53–1.51)	0.96 (0.47–1.98)
No	27 (9.4)	259 (90.6)	Reference	Reference
Farm animals				
Yes	8 (8.8)	83 (91.2)	0.99 (0.45–2.15)	
No	54 (8.9)	553 (91.1)	Reference	
Residential area				
Rural	28 (11.6)	213 (88.4)	1.64 (0.97–2.77)	
Urban	34 (7.4)	423 (92.6)	Reference	
***S. pneumoniae carriage***				
Child's nasopharynx				
Positive	58 (27.4)	154 (72.6)	45.19 (16.15–126.51)	47.30 (24.07–92.97)
Negative	4 (0.8)	480 (99.2)	Reference	Reference
Mother's nasopharynx				
Positive	5 (27.8)	13 (72.2)	4.20 (1.45–12.21)	2.34 (0.56–9.81)
Negative	57 (8.4)	623 (91.6)	Reference	Reference

*CI* confidence interval, *SD* standard deviation.

^a^Odds ratio of conjunctival *S. pneumoniae* detection for nasopharyngeal *S. pneumoniae* carriage adjusted by sex, age group (< 12 months or 12–23 months), cough, runny nose, and eye symptom (eye discharge, red eye, or itching) history in the last 2 weeks, low birth-weight, child’s day-care attendance, number of family members in the household, presence of smokers in the household, and maternal pneumococcal carriage, considering clustering in each commune.

After adjusting potential confounders, low birth-weight, day-care attendance, and having an eye symptom in the last 2 weeks independently increased *S. pneumoniae* detected in the conjunctiva (aOR 11.14, 95% CI 3.76–32.98, aOR 2.19, 95% CI 1.45–3.31, and aOR 3.59, 95% CI 2.21–5.84, respectively). We determined that *S. pneumoniae* in the nasopharynges were positively associated with *S. pneumoniae* in the conjunctivae of children (adjusted odds ratio [aOR] 47.30, 95% CI 24.07–92.97) (Table 1).

### *S. pneumoniae* serotype distribution in conjunctivae and nasopharynges

**Figure 1:**
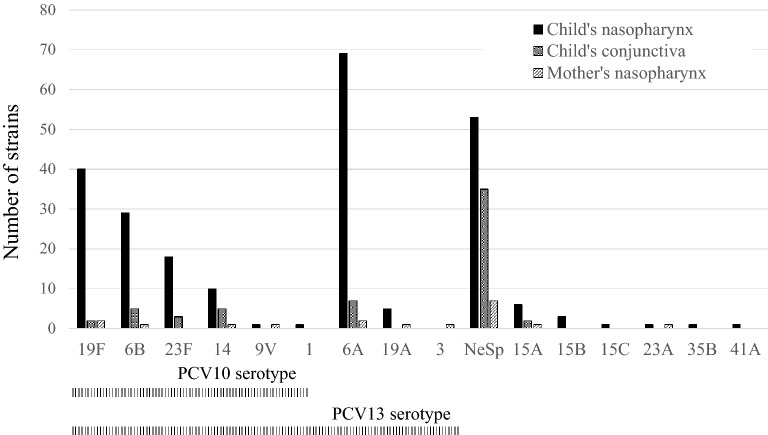
Distribution of serotype of *S. pneumoniae* detected in the child’s conjunctiva, in the child’s nasopharynx, and in the mother’s nasopharynx. NeSp; non-encapsulated *Streptococcus pneumoniae.*

The serotype was determined in 54 of 62 conjunctival samples positive for *S. pneumoniae* (87%). Five samples had two different serotypes. Of the 59 serotypes reported, non-encapsulated *S. pneumoniae* (NeSp) NT2^24^ (n = 35, 59%), 6A (n = 7, 12%), 6B (n = 5, 8%), and 14 (n = 5, 8%) were most frequently detected in the conjunctivae of children (Fig. 1). Twenty-five percent and 37% were PCV10-type and PCV13-type, respectively. Eight serotype-undetermined samples included six the autolysin-encoding gene (*lytA*)-positive with no alpha-hemolytic colony growth and two *lytA* positive with alpha-hemolytic colony growth but no *S. pneumoniae* confirmed by microarray. Two hundred and twelve children (30.5%, 95% CI 27.1–34.0%) had *S. pneumoniae* in the nasopharynx. The serotype was determined for 202 (95%) carriers; 29 (14%) had two serotypes, and 4 (2%) had three serotypes. In total, 239 serotype calls were reported, and 99 (41%) were PCV10 serotypes and 173 (72%) were PCV13 serotypes. Serotype 6A (n = 69, 28.9%) followed by NeSp (n = 53, 22.2%; NT2, n = 51; NT3b, n = 1; NT4b, n = 1), 19F (n = 40, 16.7%), 6B (n = 29, 12.1%), and 23F (n = 18, 7.5%) were the most frequently detected in the nasopharynges of children (Fig. 1). Eighteen mothers out of the 698 (2.6%, 95% CI 1.4–3.8%) had *S. pneumoniae* in the nasopharynx. The serotype was determined in 17 of the 18 mother’s nasopharyngeal samples with NeSp the most common (n = 7, 39%; NT2, n = 6; NT3b, n = 1) (Fig. 1).

Forty-seven (87%) of the 54 children with conjunctival pneumococcal detection had at least one serotype that matched with *S. pneumoniae* serotype identified in their nasopharynx. Additionally, these 47 matched serotype samples had the same genotype by array CGH analysis of the genome component of the microarray. For the five mother’s nasopharyngeal samples of children with conjunctival pneumococcal detection, three samples matched by serotype and genotype with the conjunctival and nasopharyngeal samples of their children (Table 2).

**Table 2:**
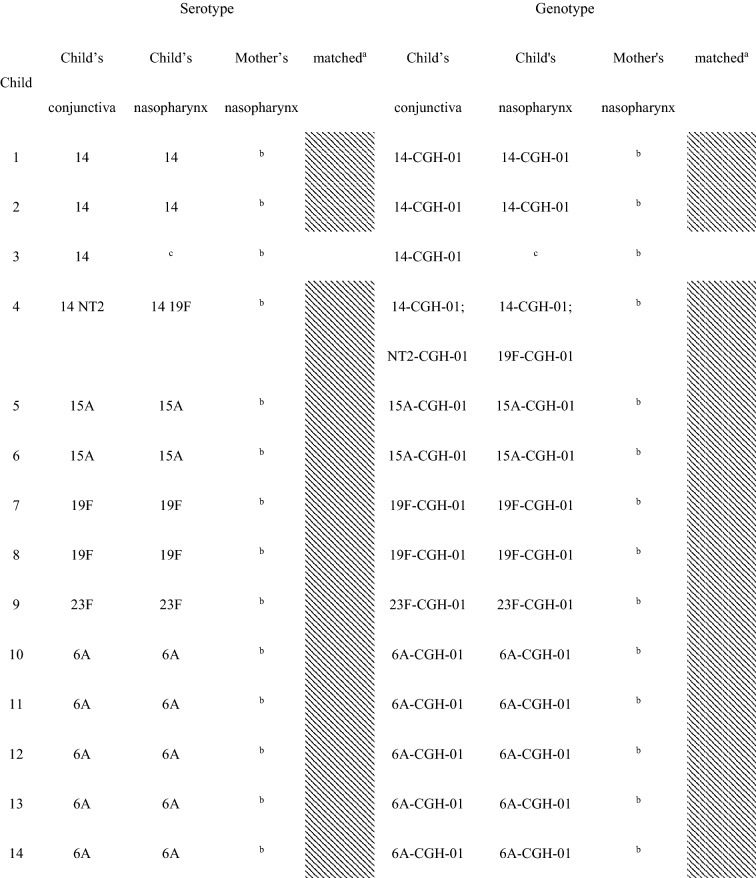

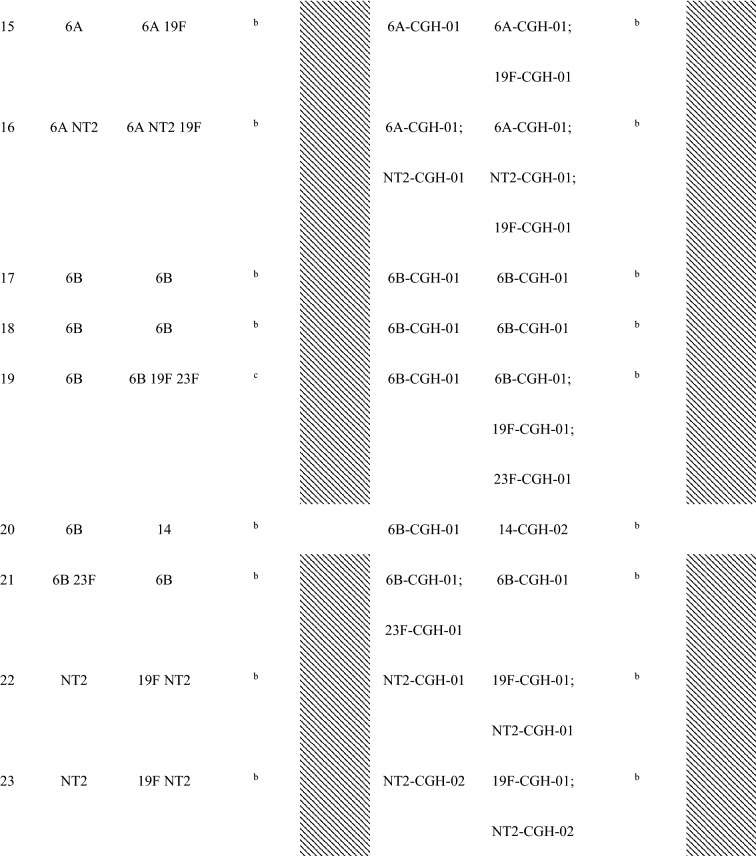

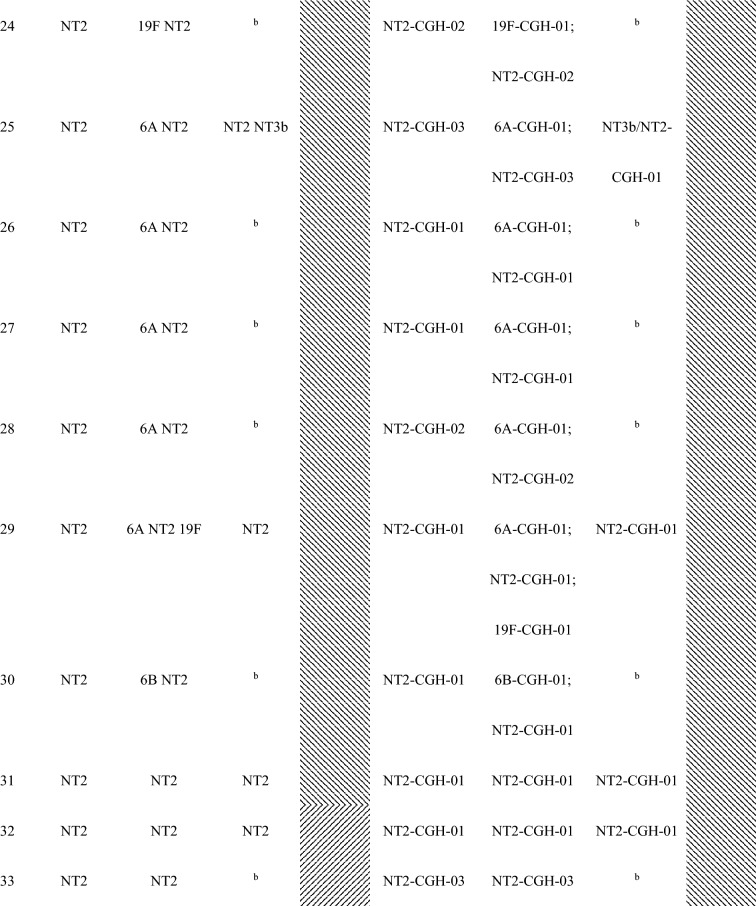

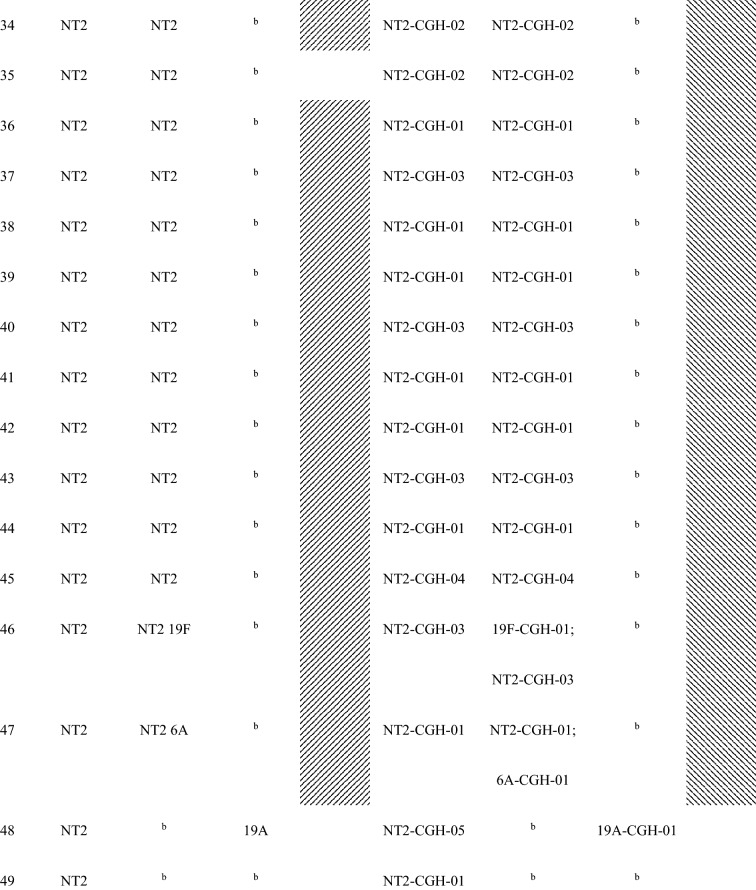

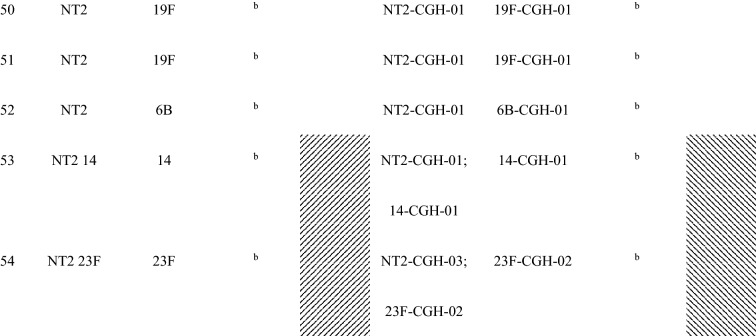
Serotypes and genotypes of *S. pnuemoniae* detected in children’s conjunctiva and nasopharynx and their mothers’ nasopharynx among children with serotype-determined conjunctival *S. pneumoniae.*

^a^Shaded had at least one serotype/MLST matched between the child’s conjunctiva and the child’s nasopharynx.

^b^*S. pneumoniae* negative.

## Discussion

To our knowledge, this study is the first to report a positive association between conjunctival *S. pneumoniae* detection and nasopharyngeal *S. pneumoniae* carriage. We conducted a large-scale community-based survey using advanced laboratory techniques for the prevalence of *S. pneumoniae* in a community.

There have been only a few studies describing the prevalence of *S. pneumoniae* in conjunctivae, which were 2.2% among people aged from 0 to more than 65 years in China^6^, 2.7% among patients awaiting cataract surgery whose mean age was 71 years in Spain^25^, 3.2% among those aged 1 to 90 in the UK^7^, and 0% in the US (no age information)^8^. In this study, the prevalence of *S. pneumoniae* in the conjunctiva among unvaccinated children aged < 24 months in a community was 8.9% (95% CI 6.9–11.2%). The higher prevalence of *S. pneumoniae* in our study may be due to several reasons including participants’ age, geography, and season or climate, considering previous reports as follows. Tao et al*.*^6^ reported a prevalence of 4.2% in children 0–6 years, and of 1.6% among those from 7 to more than 65 years old. Their results were consistent with an earlier study that found a significant difference (p < 0.002) in the prevalence of Streptococcus species in the conjunctivae of children (aged 17 years or less) and adults (14.9% versus 2.2%)^9^. The differences in *S. pneumoniae* prevalence by age, especially between adults and children, may be attributed to several potential mechanisms; including age-related changes in general immune responsiveness, tear composition and dynamics, patterns of exposure to bacteria, past antibiotic utilization, and the flora of adjacent areas, such as the skin and upper respiratory tract^9^. The difference in the prevalence of microorganisms in conjunctival flora in relation to geography was described as early as 1954 when results from eye cultures from different London based eye hospitals varied^7,26^. Seasonal variation in the same area may be another important factor. This is supported by a unique study of 4432 patients undergoing cataract surgery between 1994 and 1996 in Madrid, which showed rising isolation rates of *S. pneumoniae* in March, November, and December in ocular surface flora^25^. Also, these previous studies were conducted in temperate region and the bacteria prevalence may be different in tropical as this study. Finally, the methods for detecting *S. pneumoniae* in the conjunctiva of subjects from a community differed between the studies; while the previous studies used mostly culture techniques^6–9^, this study used highly sensitive and specific approaches, including *lytA* quantitative PCR (qPCR), culture, and DNA microarray^27^.

Previously, several studies evaluated correlations among isolates from conjunctivae, middle ear fluids, and nasopharynges in conjunctivitis-otitis syndrome^28–30^. Bingen et al*.* used restriction fragment length polymorphism to show that paired conjunctiva and middle ear fluid isolates of *Haemophilus influenzae* were identical^29^. Groothuis et al. explored the correlation between conjunctival and nasopharyngeal cultures among conjunctivitis-otitis media syndrome^30^. However, there have been no studies demonstrating the association of *S. pneumoniae* in conjunctivae and nasopharynges in members of a community. Here, we report the association between *S. pneumoniae* in the conjunctiva and the nasopharynx.

*S. pneumoniae* is a causative agent of many documented conjunctivitis outbreaks, and NT pneumococci are commonly identified as the etiological agent^31–33^. The association of NT *S. pneumoniae* with conjunctivitis was first suggested by Finland and Barnes in 1977^32^. Indeed, NT *S. pneumoniae* have caused several conjunctivitis outbreaks in the USA^34,35^, and were also recognized as a frequent cause of sporadic cases of conjunctivitis in Spain^22^. Previous studies found that 23–90% of *S. pneumoniae* isolates causing conjunctivitis were NT, with higher rates after PCV programs were introduced^22,31,33^. In studies using phenotypic testing, NTs may include both non-encapsulated *S. pneumoniae* as well as isolates for which no serotype was determined (e.g. due to the isolate not expressing capsule, or the test not including all serotypes). Keller et al*.* emphasized that surface proteins unique to non-encapsulated *S. pneumoniae* enhanced colonization and virulence despite the lack of a capsule^33^. Our study determined that 59% of *S. pneumoniae*-positive conjunctival samples were NeSp, the majority of which were NT2 that includes the NspA/PspK surface protein encoded by the null capsule locus^24^. Besides, 37% of PCV13-type *S. pneumoniae* might imply the effect of PCV introduction on reducing the prevalence in conjunctiva and also incidence of pneumococcal conjunctivitis in this area.

Although *S. pneumoniae* in nasopharynx and conjunctiva showed concordance in serotype and genotype among matched samples from children, the serotype distribution in the two sites was different. The most frequent conjunctival serotypes in this study were NeSp, 6A, 6B, 14, 23F, and 19F, among which NeSp had a markedly high (59%) and 6A had low (12%) proportion compared to those in the nasopharynx (22% and 29%, respectively) among the same subjects. Specific conjunctival factors, including tissue immune responsiveness, tear composition (immunoglobulins and antimicrobial enzyme lysozyme), dynamics (mechanical action of the eyelids), and patterns of exposure to bacteria might have an important role in serotype distribution differences between the conjunctiva and the nasopharynx^9,25^.

In our study, the lower rate of conjunctival *S. pneumoniae* detection than nasopharyngeal carriage, and the concordance of serotype and genotype in matched samples, might indicate that colonization of *S. pneumoniae* starts in the nasopharynx and spreads to the conjunctiva. Also, the tear composition and dynamics explained above might make it more difficult for potentially pathogenic microorganisms to colonize the conjunctival surface^9^. *S. pneumoniae* can be plausibly transmitted from the nasopharynx to the conjunctiva through the nasolacrimal duct, by the retrograde passage of fluid from the nose to the conjunctiva during nasal congestion and because of short ducts during infancy and early childhood. Also, *S. pneumoniae* may reach the conjunctival sac from the skin, the surrounding environment, hand contamination, and through contact with persons such as the mother^36^. The low nasopharyngeal carriage prevalence of mothers in this study indicates that they were unlikely to be the major source of transmission. We did not investigate the conjunctival *S. pneumoniae* prevalence in mothers in this study; however, it would be interesting to consider the same in a future study with a larger sample size to determine the prevalence in mothers and the potential transmission route.

To our knowledge, this is the first study to investigate risk factors related to conjunctival *S. pneumoniae* detection in children. Whilst we anticipated that age would be a risk factor for conjunctival *S. pneumoniae* detection, we did not find a difference in our study, most likely because the paediatric groups were relatively similar in age. Day-care attendance increased conjunctival *S. pneumoniae* detection in this study. Day-care attendance is a well-established factor associated with nasopharyngeal *S. pneumoniae* carriage^16,19,37,38^. Our results are in accordance with Sthapit et al*.*, who showed that conjunctival bacterial flora is similar in either sex^39^. This study also determined that low birth-weight increased *S. pneumoniae* detection in the conjunctiva in children < 24 months. This may be because of the susceptibility of low birth-weight children to infectious diseases, compared to normal birth weight children, due to poor immune responses^40^. In contrast, the decreased nasopharyngeal carriage risk with low birth-weight was a surprising finding, though this result has been reported in Dutch and Indian babies^19,41^.

There are some limitations to the study. First, this was a cross-sectional study conducted in October 2016, and results may vary by season. Longitudinal studies may also provide insight as to whether detection of *S. pneumoniae* in the conjunctiva of children in a community is a true carriage, such as observed in the nasopharynx. In a few cases, the prevalence of pneumococcal strains was determined by positive *lytA* detection only. It should be considered that other related streptococcal species may also have *lytA* homologues and could yield false-positive results. Nevertheless, this accounted for a minor proportion, which would not have affected the findings of this study. In addition, we did not assess the presence of viruses or other bacterial species in this study.

Nearly 9% of children in a central Vietnamese community aged < 24 months had *S. pneumoniae* in the conjunctiva before introduction of PCV in the community. We have reported the novel finding that *S. pneumoniae* in the conjunctiva was positively associated with that in the nasopharynx, and that the serotypes and genotypes in the conjunctiva mostly matched those in the nasopharynx for each child. These findings suggest that the nasopharynx may be a source of transmission for *S. pneumoniae* to the conjunctiva. Nevertheless, there was some evidence also for a different serotype distribution in nasopharynx and in conjunctiva of study subjects. In addition, there was a high proportion of NeSp in the conjunctiva. Our data provide further evidence that NeSp has a unique ability to colonize in the ocular environment.

## Methods

### Study site and participants

The study was conducted in six communes in the city of Nha Trang, central Vietnam. Nha Trang has 27 communes and each commune has one commune health center, providing a range of basic health services. In the study area, two communes were zoned as rural and four as urban areas, by the administrative boundary. A PCV-reduced dosing schedule trial has been initiated in this study site^42^, and the current study was a part of the pre-PCV baseline assessment of the trial (https://clinicaltrials.gov/ct2/show/NCT02961231).

In each commune, 60 children, each from two target age groups, aged 4–11 months (younger group) and 14–23 months (older group), were randomly selected using the commune population registration records for enrolment into the study, regardless of current respiratory or eye symptoms. The populations of target age groups in this study, within the study area of the 6 communes, were 1004 for 4–11 months and 1590 for 14–23 months. The randomly selected children and their mothers were invited for examination and interview at the commune health center in each commune, in October 2016, before the introduction of PCV. Written informed consent was obtained from all parents or guardians before collecting samples and conducting interviews.

### Data, sample collection, and testing

A trained healthcare worker in each commune health center interviewed each participant’s mother to collect demographic, socioeconomic, and clinical information using a structured interview form. Data collected included: sex, date of birth, birth-weight, gestational age at birth, if the child has ever been hospitalized for respiratory disease, PCV history, current symptoms in the last 2 weeks (cough, runny nose, eye symptoms including eye discharge, red eye, itchy eye, and others), breast feeding history, child’s day-care attendance, whether the child is usually living with other children aged under five, education level of the mother, household income last month, family members smoking, household having farm animals, and residential area. Births were defined as preterm if children were born < 37 gestational weeks, and of low birth-weight if the birth-weight was < 2500 g, following the guidelines in the International Classification of Diseases-10: version 2010^43^. The child’s conjunctival and nasopharyngeal swabs, and the mother’s nasopharyngeal swabs, were obtained by a doctor using a nylon flocked swab and stored in skim milk, tryptone, glucose, and glycerol (STGG) medium according to WHO guidelines^44^.

Swabs were sent to the Pasteur Institute Nha Trang, where DNA extraction and real-time qPCR targeting *lytA* of *S. pneumoniae* was performed. Samples that were positive (Ct value < 35) or equivocal (Ct value 35–40) by qPCR-testing were cultured on selective agar, and DNA was extracted from bacterial growth on the QIAcube HT (Qiagen, Hilden, Germany) platform as previously described^45^. The extracted DNA was sent to the Murdoch Children's Research Institute, Melbourne, Australia, for molecular serotyping by microarray (Senti-SP v1.5, BUGS Bioscience, London, UK; http://bugsbio.org) to determine the serotype and genotype of *S. pneumoniae* present in the conjunctival and nasopharyngeal samples. Pneumococcal carriage and the presence of each pneumococcal serotype were determined by microarray. Samples that were *lytA* qPCR positive (Ct value < 35) but not able to be serotyped (either culture negative or low DNA yield from culture) were considered pneumococcal positive, serotype unknown^45^.

### Sample size

Sample size was calculated based on the primary outcome of presence of conjunctival *S. pneumoniae.* We assumed the true proportion of children aged < 24 months in the study area who have *S. pneumoniae* in the conjunctiva to be 0.5 to obtain the largest sample size, and required the estimate to be within 0.04 of the true value (precision) within the 95% confidence interval (CI). Then, the minimum sample size was calculated to be 600.

### Ethical considerations

Institutional Review Boards at the National Institute of Hygiene and Epidemiology, Hanoi, Vietnam (the approval number VN01057), and the Institute of Tropical Medicine, Nagasaki University, Nagasaki, Japan (151203149-2), approved this study. All methods were performed in accordance with the relevant guidelines and regulations.

### Statistical analysis

We calculated the prevalence of conjunctival and nasopharyngeal *S. pneumoniae* among children < 24 months and that of nasopharyngeal *S. pneumoniae* among their mothers in the study area. *S. pneumoniae* serotype distribution in the conjunctiva and nasopharynx were shown graphically and the concordance was evaluated. We showed the number and the proportion of children with the same *S. pneumoniae* serotype/genotype in the nasopharynx and in the conjunctiva. We compared demographic, socioeconomic, and clinical characteristics, and *S. pneumoniae* prevalence in the nasopharynges of the children and mothers between children with and without conjunctival *S. pneumoniae.* Crude odds ratios of conjunctival *S. pneumoniae* detection were analyzed for each characteristic using logistic regression. Based on previous studies of risk factors associated with conjunctival bacterial prevalence^6,39^ or nasopharyngeal *S. pneumoniae* carriage^16,19,46,47^, the following potential confounders were selected a priori and included in a model to estimate the effect of the child’s nasopharyngeal *S. pneumoniae* carriage on conjunctival *S. pneumoniae* detection: sex, age group (< 12 months or 12–23 months), cough, runny nose, and eye symptoms (eye discharge, red eye, or itching) history in the last 2 weeks, low birth-weight, day-care attendance, number of family members in the household, presence of smokers in the household, and maternal pneumococcal carriage. CIs were adjusted for the clustering of communes using robust standard errors. Statistical analyses were conducted using Stata version 14.0 software (StataCorp. 2015. Stata Statistical Software: Release 14. College Station, TX: StataCorp LP).

## Data Availability

The datasets generated during and/or analysed during the current study are available from the corresponding author on reasonable request.

## References

[1] Armstrong RA (2000). The microbiology of the eye. Ophthal. Physiol. Opt..

[2] Deorukhkar S, Katiyar R, Saini S (2012). Epidemiological features and laboratory results of bacterial and fungal keratitis: A five-year study at a rural tertiary-care hospital in western Maharashtra, India. Singapore Med. J..

[3] Orden Martínez B, Martínez Ruiz R, Millán Pérez R (2004). Bacterial conjunctivitis: Most prevalent pathogens and their antibiotic sensitivity. An. Pediatr..

[4] Block SL (2000). Increasing bacterial resistance in pediatric acute conjunctivitis (1997–1998). Antimicrob. Agents Chemother..

[5] Gigliotti F (1981). Etiology of acute conjunctivitis in children. J. Pediatr..

[6] Tao H (2017). Incidence and antimicrobial sensitivity profiles of normal conjunctiva bacterial flora in the central area of China: A hospital-based study. Front. Physiol..

[7] Smith CH (1954). Bacteriology of the healthy conjunctiva. Br. J. Ophthalmol..

[8] Perkins RE, Kundsin RB, Pratt MV (1975). Bacteriology of normal and infected conjunctiva. J. Clin. Microbiol..

[9] Singer TR, Isenberg SJ, Apt L (1988). Conjunctival anaerobic and aerobic bacterial flora in paediatric versus adult subjects. Br. J. Ophthalmol..

[10] Wahl B (2018). Burden of *Streptococcus**pneumoniae* and *Haemophilus**influenzae* type b disease in children in the era of conjugate vaccines: Global, regional, and national estimates for 2000–15. Lancet Glob. Health.

[11] Peltola H (2001). Burden of meningitis and other severe bacterial infections of children in Africa: Implications for prevention. Clin. Infect. Dis..

[12] Ayieko P, English M (2007). Case management of childhood pneumonia in developing countries. Pediatr. Infect. Dis. J..

[13] Lieberthal AS (2013). The diagnosis and management of acute otitis media. Pediatrics.

[14] Slavin RG (2005). The diagnosis and management of sinusitis: A practice parameter update. J. Allergy Clin. Immunol..

[15] Buznach N, Dagan R, Greenberg D (2005). Clinical and bacterial characteristics of acute bacterial conjunctivitis in children in the antibiotic resistance era. Pediatr. Infect. Dis. J..

[16] Bogaert D, de Groot R, Hermans PWM (2004). *Streptococcus**pneumoniae* colonisation: The key to pneumococcal disease. Lancet Infect. Dis..

[17] Simell B (2012). The fundamental link between pneumococcal carriage and disease. Expert Rev. Vaccines.

[18] Raman R, Sankar J, Putlibai S, Raghavan V (2017). Demographic profile of healthy children with nasopharyngeal colonisation of *Streptococcus**pneumoniae*: A research paper. Indian J. Med. Microbiol..

[19] Labout JAM (2008). Factors associated with pneumococcal carriage in healthy Dutch infants: The generation R study. J. Pediatr..

[20] Sugita G (2014). Genetic characteristics of *Haemophilus**influenzae* and *Streptococcus**pneumoniae* isolated from children with conjunctivitis-otitis media syndrome. J. Infect. Chemother..

[21] Bodor FF, Marchant CD, Shurin PA, Barenkamp SJ (1985). Bacterial etiology of conjunctivitis-otitis media syndrome. Pediatrics.

[22] Berrón S, Fenoll A, Ortega M, Arellano N, Casal J (2005). Analysis of the genetic structure of nontypeable pneumococcal strains isolated from conjunctiva. J. Clin. Microbiol..

[23] Marimon JM, Ercibengoa M, García-Arenzana JM, Alonso M, Pérez-Trallero E (2013). *Streptococcus**pneumoniae* ocular infections, prominent role of unencapsulated isolates in conjunctivitis. Clin. Microbiol. Infect..

[24] Salter SJ (2012). Variation at the capsule locus, cps, of mistyped and non-typable *Streptococcus**pneumoniae* isolates. Microbiology.

[25] Rubio EF (2004). Climatic influence on conjunctival bacteria of patients undergoing cataract surgery. Eye.

[26] Grzybowski A, Brona P, Kim SJ (2017). Microbial flora and resistance in ophthalmology: A review. Graefe. Arch. Clin. Exp. Ophthalmol..

[27] Satzke C (2015). The PneuCarriage Project: A multi-centre comparative study to identify the best serotyping methods for examining pneumococcal carriage in vaccine evaluation studies. PLoS Med..

[28] Bodor FF (1998). Diagnosis and management of acute conjunctivitis. Semin. Pediatr. Infect. Dis..

[29] Bingen E, Cohen R, Jourenkova N, Gehanno P (2005). Epidemiologic study of conjunctivitis–otitis syndrome. Pediatr. Infect. Dis. J..

[30] Groothuis JR, Thompson J, Wright PF (1986). Correlation of nasopharyngeal and conjunctival cultures with middle ear fluid cultures in otitis media: A prospective study. Clin. Pediatr..

[31] Williamson YM (2008). Differentiation of *Streptococcus**pneumoniae* conjunctivitis outbreak isolates by matrix-assisted laser desorption ionization-time of flight mass spectrometry. Appl. Environ. Microbiol..

[32] Finland M, Barnes MW (1977). Changes in occurrence of capsular serotypes of *Streptococcus**pneumoniae* at Boston City Hospital during selected years between 1935 and 1974. J. Clin. Microbiol..

[33] Keller, L. E., Robinson, D. A. & McDaniel, L. S. Nonencapsulated *Streptococcus**pneumoniae*: Emergence and pathogenesis. *mBio* **7**, e01792–15. https://mbio.asm.org/content/7/2/e01792-15 (2016). 10.1128/mBio.01792-15 PMC4807366 27006456

[34] Crum NF, Barrozo CP, Chapman FA, Ryan MAK, Russell KL (2004). An outbreak of conjunctivitis due to a novel unencapsulated *Streptococcus**pneumoniae* among military trainees. Clin. Infect. Dis..

[35] Martin M (2003). An outbreak of conjunctivitis due to atypical *Streptococcus**pneumoniae*. N. Engl. J. Med..

[36] Mejía-López H, Pantoja-Meléndez CA, Climent-Flores A, Bautista-de Lucio VM, Pelikan Z (2011). Epidemiological aspects of infectious conjunctivitis. Conjunctivitis—A Complex and Multifaceted Disorder.

[37] Ghaffar F, Friedland IR, McCracken GH (1999). Dynamics of nasopharyngeal colonization by *Streptococcus**pneumoniae*. Pediatr. Infect. Dis. J..

[38] Regev-Yochay G (2004). Association between carriage of *Streptococcus**pneumoniae* and *Staphylococcus**aureus* in children. J. Am. Med. Assoc..

[39] Sthapit PR, Tuladhar NR (2014). Conjunctival flora of normal human eye. JSM Ophthalmol..

[40] Soto-Noguerón A (2016). *Streptococcus**pneumoniae* as cause of infection in infants less than 60 days of age: Serotypes and antimicrobial susceptibility. Int. J. Infect. Dis..

[41] Coles CL (2001). Pneumococcal nasopharyngeal colonization in young South Indian infants. Pediatr. Infect. Dis. J..

[42] Nagasaki University. Evaluation of PCV schedules in a naive population in Vietnam. https://clinicaltrials.gov/ct2/show/NCT02961231 (2016).

[43] World Health Organization. ICD-10 Version:2010. http://apps.who.int/classifications/icd10/browse/2010/en (2010).

[44] Satzke C (2013). Standard method for detecting upper respiratory carriage of *Streptococcus**pneumoniae*: Updated recommendations from the World Health Organization Pneumococcal Carriage Working Group. Vaccine.

[45] Satzke C (2019). Pneumococcal carriage in vaccine-eligible children and unvaccinated infants in Lao PDR two years following the introduction of the 13-valent pneumococcal conjugate vaccine. Vaccine.

[46] Usuf E (2018). Maternal pneumococcal nasopharyngeal carriage and risk factors for neonatal carriage after the introduction of pneumococcal conjugate vaccines in The Gambia. Clin. Microbiol. Infect..

[47] García-Rodríguez JA, Fresnadillo Martínez MJ (2002). Dynamics of nasopharyngeal colonization by potential respiratory pathogens. J. Antimicrob. Chemother..

